# VEGF expression by mesenchymal stem cells contributes to angiogenesis in pancreatic carcinoma

**DOI:** 10.1038/sj.bjc.6604508

**Published:** 2008-07-29

**Authors:** B M Beckermann, G Kallifatidis, A Groth, D Frommhold, A Apel, J Mattern, A V Salnikov, G Moldenhauer, W Wagner, A Diehlmann, R Saffrich, M Schubert, A D Ho, N Giese, M W Büchler, H Friess, P Büchler, I Herr

**Affiliations:** 1 Molecular OncoSurgery Group, Department of General Surgery, University of Heidelberg and German Cancer Research Center, Heidelberg, Germany; 2 Department of General Surgery, University of Heidelberg, Heidelberg, Germany; 3 Department of Neonatology, University of Heidelberg, Heidelberg, Germany; 4 Department of Molecular Immunology, German Cancer Research Center, Heidelberg, Germany; 5 Department of Medicine V, University of Heidelberg, Heidelberg, Germany; 6 Department of General Surgery, Klinikum rechts der Isar, Technische Universität München, Munich, Germany

**Keywords:** MSC, angiogenesis, lentivirus, VEGF, pancreas

## Abstract

Little is known about the factors that enable the mobilisation of human mesenchymal stem cells (MSC) from the bone marrow into the blood stream and their recruitment to and retention in the tumour. We found specific migration of MSC towards growth factors present in pancreatic tumours, such as PDGF, EGF, VEGF and specific inhibitors Glivec, Erbitux and Avastin interfered with migration. Within a few hours, MSC migrated into spheroids consisting of pancreatic cancer cells, fibroblasts and endothelial cells as measured by time-lapse microscopy. Supernatant from subconfluent MSC increased sprouting of HUVEC due to VEGF production by MSC itself as demonstrated by RT-PCR and ELISA. Only few MSCs were differentiated into endothelial cells *in vitro*, whereas *in vivo* differentiation was not observed. Lentiviral GFP-marked MSCs, injected in nude mice xenografted with orthotopic pancreatic tumours, preferentially migrated into the tumours as observed by FACS analysis of green fluorescent cells. By immunofluorescence and intravital microscopic studies, we found the interaction of MSC with the endothelium of blood vessels. Mesenchymal stem cells supported tumour angiogenesis *in vivo*, that is CD31^+^ vessel density was increased after the transfer of MSC compared with siVEGF-MSC. Our data demonstrate the migration of MSC toward tumour vessels and suggest a supportive role in angiogenesis.

The plastic adherent cells from the bone marrow (BM) referred to as mesenchymal stem cells (MSC) are capable of self-renewing and have the potential to differentiate into mesenchymal and non-mesenchymal tissues (Prockop, 1997). Mesenchymal stem cells contribute to tissue regeneration by differentiation into bone, cartilage, muscle, ligament, tendon, adipose tissue, and stroma (Pittenger *et al*, 1999). The ability of MSC to migrate to the areas of injury and to tumours has encouraged investigation of MSC as therapeutic tools. For example, systemically administered MSCs have been shown to improve recovery in animal models of stroke and myocardial infarction (Mahmood *et al*, 2003; Fukuda and Fujita, 2005). Mesenchymal stem cells have also been used for targeted delivery of therapeutic gene products to the tumour microenvironment in animal models (Studeny *et al*, 2002, 2004; Nakamizo *et al*, 2005). This shared tropism of MSC for sites of injured tissue and for tumours is believed to result from similarities in the inflammatory milieu produced by healing wounds and tumours, evoking the notion that ‘tumours are wounds that never heal’ (Dvorak, 1986). Furthermore, a role of MSC in neoangiogenesis is discussed, as the administration of MSC stimulated revascularisation of ischaemic tissues (Chen *et al*, 2003; Nagaya *et al*, 2004). A correlation between angiogenesis in melanoma and MSC has recently been demonstrated (Sun *et al*, 2005), and MSC have been found to transmigrate over the endothelial barrier (Schmidt *et al*, 2006b). In all cases, MSCs had to cover a distance to reach the target area. Therefore, the common hypothesis is that MSC possess a migratory activity. The most prominent chemotactic factors identified thus far for MSC include SDF-1, basic fibroblast growth factor and vascular endothelial growth factor (VEGF) (Schmidt *et al*, 2006a). The concept of chemokines as possible chemoattractants for MSC may be of importance for pancreatic cancer, as this tumour entity contains poorly vascularised regions characterised by severe hypoxia resulting in the expression of growth factors (Bos *et al*, 2005; Patiar and Harris, 2006).

It has been suggested that the MSC-mediated effects can be attributed, at least in part, to the biologically active factors secreted by MSC itself at their target sites. Even more, a direct contribution of MSC to the blood vessel formation is suggested, as differentiation of MSC into endothelial cells has been demonstrated (Oswald *et al*, 2004; Silva *et al*, 2005; Song *et al*, 2007), although this concept remains controversial. Here, we test the hypothesis that hypoxia-induced growth factor expression in pancreatic cancer promotes tumour angiogenesis by mediating MSC recruitment.

We assessed this hypothesis by *in vitro* studies and in an orthotopic mouse model of pancreatic carcinoma. We demonstrate the migration of MSC towards growing normal and tumour cells, as well as to platelet-derived growth factor (PDGF), epidermal growth factor (EGF), and vascular epidermal growth factor (VEGF). Inhibitors of PDGFR (Glivec), EGFR (Erbitux) and blocking antibody to VEGF (Avastin) interfered with MSC migration demonstrating the specific growth factor-mediated effect. Within a few hours, MSC migrated into pancreatic tumour cell spheroids as measured by time-lapse microscopy. Mesenchymal stem cells themselves secreted VEGF, and the transfer of supernatant from cultured MSC induced sprouting of endothelial cells. Differentiation of MSC to endothelial cells was observed in only few cells *in vitro* but not *in vivo*. However, MSC inoculated in nude mice xenografted with orthotopic pancreatic tumours were found to home into the tumours and to incorporate in tumour blood vessels. Our conclusion is that MSC contribute to the tumour blood vessel formation by homing to fast growing tumours and the incorporation into blood vessels as atypical VEGF-secreting endothelial cells.

## Materials and methods

### Isolation and culture of human BM-derived MSC

Mesenchymal stem cells were isolated from human BM of healthy donors, selected by plastic adherence, and were cultivated as described in our recent publication (Kallifatidis *et al*, 2008).

### Primary and established human cell lines

Established human standard cancer cell lines of pancreas (Capan-1, MIA-Pa-Ca2, Colo-357, and BxPC-3), immortalised human kidney cells (HEK 293T), and human primary fibroblasts from the skin (kind gift from J Knebel and P Angel) were cultured in DMEM supplemented with 10% FCS. Human umbilical vein endothelial cells (HUVEC; PromoCell, Heidelberg, Germany) were cultured in endothelial cell growth medium (ECGM; PromoCell).

### Migration of MSC to chemoattractants measured by transwell chamber migration assay

A ChemoTx® System (Neuro Probe Inc., Gaithersburg, MD, USA) with 96 wells or Transwell® permeable supports (Corning Incorporated, Life Sciences, Acton, MA, USA) and a polycarbonate membrane pore size of 12 *μ*m was used. The bottom chamber contained PDGF (R&D Systems, Wiesbaden-Nordenstadt, Germany), EGF (R&D Systems), or VEGF (BioSource, Nivelles, Belgium) in medium with 2% FCS or cell culture supernatant of tumour cells grown in medium supplemented with 2% FCS for 2 days. Growth factors were used with or without specific inhibitors: Avastin for the inhibition of VEGF (25 *μ*g ml^−1^; Roche, Welwyn Garden City, Hertfordshire, UK), Glivec for the inhibition of PDGF receptors (3 *μ*M; Novartis, Horsham, West Sussex, UK), and Erbitux for the inhibition of EGF receptors (3 *μ*M; Merck, Darmstadt, Germany). The migration of MSC was analysed as described in our recent publication (Kallifatidis *et al*, 2008).

### Generation of tumour cell spheroids

Spheroids were generated as described (Korff *et al*, 2004). Confluent monolayers of MIA-PaCa-2 cells, primary fibroblasts, and HUVEC were trypsinised. A total of 500 MIA-PaCa-2 cells, 250 fibroblasts, and 250 HUVECs per spheroid were mixed in corresponding culture medium containing 0.25% (w/v) methylcellulose (Sigma, Steinheim, Germany) and seeded in nonadherent round bottom 96-well plates (Greiner, Frickenhausen, Germany). Under these conditions, all suspended cells contribute to the formation of a single spheroid per well of defined size and cell number. Spheroids were cultured for at least 24 h and used for the corresponding assay.

### Migration of MSC to tumour cell spheroids

Mesenchymal stem cells were labelled with CellTracker Red according to the instructions of the manufacturer (Molecular Probes, Eugene, OA, USA). A total of 1 × 10^4^ MSCs (in a volume of 225 *μ*l) were seeded in one edge of a fibronectin-coated well of a 24-well plate by canting the plate in an angle of 30°. After 24 h, MSC culture medium was removed, and cells were covered with 225 *μ*l of methylcellulose/collagen solution (40% methylcellulose stock solution, 10% FCS, and 50% collagen solution prepared from rat tail). Correspondingly, spheroids were labelled with Cell Tracker Green (Molecular Probes), and 48 spheroids were seeded opposite to the MSC in the same well. Invasion of MSC in spheroids was documented by time-lapse microscopy and a camera with a red filter and green filter. The camera was focused to the green fluorescent spheroids and cells were observed over 12 h on an Olympus IX70 microscope equipped with an incubation housing around the microscope. Time-lapse imaging series were acquired using the software analySiS from Soft Imaging System with a ColorView-12 digital colour camera. Images were made every 5 min, for each position one with bright field and one with fluorescence illumination in red and/or green with a motorised Ludl X-Y microscope stage.

### *In vitro* angiogenesis assay

Spheroids containing 750–1000 HUVECs were generated overnight, after which they were embedded in collagen gel as described previously (Korff *et al*, 2001). Supernantant of MSC or recombinant VEGF (BioSource) were transferred to the spheroids. After 24 h, *in vitro* angiogenesis was digitally quantified by measuring the length of the sprouts that had grown out of each spheroid (at × 10 magnification) using the digital imaging software cell^B^ 2.3 (Olympus, Hamburg, Germany) analysing at least eight spheroids per experimental group and experiment.

### Detection of VEGF and *α*-smooth muscle actin in MSC by RT-PCR

Mesenchymal stem cells were seeded in a six-well plate at a concentration of 1 × 10^5^ cells per well and incubated overnight under normoxic conditions (37°C, 5% CO_2_) followed by 16 h incubation in a hypoxia chamber (N_2_: 89.25%, CO_2_: 10%, and O_2_: 0.75%). Cells were scraped in 300 *μ*l per well prewarmed Magna Pure LC Lysis buffer (mRNA isolation kit I for cells; Roche Applied Science, RAS, Mannheim, Germany) supplemented with 0.01 g ml^−1^ DTT from the tissue culture plates and frozen at −80°C. mRNA/cDNA preparation and real-time quantitative PCR were performed with equipment and reagents from Roche as described previously (Erkan *et al*, 2007). In brief, mRNA was extracted by automated isolation, and cDNA was prepared using the First Strand cDNA synthesis kit for RT-PCR (ABgene Advanced Biotechnologie, Epsom, UK). QRT-PCR was performed using LightCycler™ primer sets obtained from Search-LC (Heidelberg, Germany). Results are expressed as the number of VEGF or *α*-SMA transcripts per 10 000 CPB transcripts (cyclophilin B, a housekeeping gene).

### Lentiviral transduction of MSC

Vectors used in our study are the self-inactivating (SIN) vectors of the second generation, which loose the activity of the promoter located in the 5′-LTR upon replication and integration into the genome of the host cells. The construction of the pLL3.7puroeGFP vector plasmid, VEGF siRNA, and lentiviral transduction of MSC is described in our recent manuscript (Kallifatidis *et al*, 2008). In detail, the VEGF siRNA sequence was designed against the human VEGF mRNA (accession no. AF022375), the target sequence starting at position 465 after the start codon. Depicted is the resulting VEGF siRNA sequence with polyA tail, siRNA stem, loop and siRNA stem: 5′-tcgagaaaaaagatccgcagacgtgtaaatgtctcttgaacatttacacgtctgcggatca-3′. The construct was verified by sequencing.

### Detection of VEGF in cell culture supernatant of MSC

A volume of 5 × 10^4^/ml lentiviral transduced or non-transduced MSC were seeded in 12-well plates. Hypoxia was induced as described above, and 16 h later, cells were quickly removed from hypoxic conditions by putting them on ice. The VEGF content was estimated in supernatant and cell lysates following the instructions of the Quantikine® ELISA for human VEGF (R&D Systems, Wiesbaden-Nordenstadt, Germany). Cells treated with 25 *μ*g ml^−1^ Avastin added at the time of hypoxia induction served as negative control. Vascular endothelial growth factor expression was evaluated in an ELISA plate reader at 450 nm with a correction at 570 nm. Results were normalised to picogram VEGF per hour treatment per 10^4^ cells.

### *In vitro* differentiation of MSC in endothelial cells

Mesenchymal stem cells (1 × 10^4^/cm^2^) were seeded in a six-well plate, and for differentiation, 50 ng ml^−1^ VEGF (Biosource, Nivelles, Belgium) was added to standard culture medium or to ECGM used for HUVEC culture. Differentiation to endothelial cells was analysed by using the Chemicon (Temecula, CA, USA) blood vessel staining kit following supplier's instructions. Shortly, the cells were incubated with rabbit anti-vWF polyclonal antibody (1 : 200, Chemicon) or mouse anti-CD31 monoclonal antibody (1 : 200, Chemicon) and detected with biotinylated goat anti-rabbit or goat anti-mouse antibody and Streptavidin-HRP (Chemicon). DAB/haematoxylin staining was performed by a standard protocol. Cells were analysed with a Leica DMRB microscope (Leica Microsystems GmbH, Wetzlar, Germany) with Kappa CF20/4 DX Camera (Kappa Opto-Electronics GmbH, Gleichen, Germany).

### Detection of microvessel density in xenografts

To examine the effects of MSC injection on the microvessel density in xenografts, aceton-fixed frozen sections (5 *μ*m) were stained with rat anti-mouse CD31 mAb (PharMingen, San Diego, CA, USA) as described previously (30). Random areas of tumours were then examined under higher magnification ( × 250) and CD31^+^ structures were counted. Any distinct area of positive staining for CD31 was counted as a single vessel. Results were expressed as the mean number of vessels±s.e. per mm^2^. A total of eight high-power fields was examined and counted from four tumours of each of the treatment groups.

### Orthotopic pancreatic cancer xenograft model in athymic nude mice

NMRI (nu/nu) male mice (6-to-10-weeks old) were used for subcutaneous and orthotopic tumour implantations of the human pancreatic cancer cell line MIA-PaCa-2 as described previously (Buchler *et al*, 2007). The experimental protocol was approved by the Chancellor's Animal Research Committee of the University of Heidelberg (Heidelberg, Germany) in accordance with National Guidelines for Animal Care and the Use of Laboratory Animals.

### Detection of GFP-MSC in blood vessels of pancreatic cancer xenografts

Cryosections were fixed in 4% PFA for 15 min and permeabilised in 0.2% Triton X-100 for 15 min. The sections were incubated with mouse anti-eGFP antibody 1 : 200 (JL8, BD Clontech, Heidelberg, Germany) in PBS/5% goat serum and detected with goat anti-mouse FITC-conjugated antibody (Invitrogen, Karlsruhe, Germany) diluted 1 : 200 in PBS/5% goat serum. For secondary staining, sections were incubated with rabbit anti-vWF antibody 1 : 200 (Chemicon) in PBS/5% goat serum and detected with biotinylated goat anti-rabbit Ab 1 : 200 (KPL, Gaithersburg, MD, USA) and Texas Red Avidin 1 : 200 (Vector Laboratories, Peterborough, UK).

### Intravital microscopy of GFP-MSC recruitment in mice

Anaesthesia, general preparation, surgical preparation of the cremaster muscle and xenograft, intravital microscopy, and data analysis were performed as previously described (Sperandio *et al*, 2001, 2006). Briefly, after anaesthesia, the trachea was intubated, and the left carotid artery was cannulated with PE 10 tubing (ID: 0.28 mm, OD: 0.61 mm; Becton Dickinson) for the administration of MSC and anaesthetics throughout the intravital microscopic experiment. For prevention of ischaemic events, the number of injected cells was restricted to 1 × 10^5^ MSC/0.2 ml normal saline per application up to a total number of 4 × 10^5^ MSC during 1 h. Intravital microscopy was conducted on an upright microscope (Leitz, Wetzlar, Germany) with a saline immersion objective (SW 40/0.75 numerical aperture) and epifluorescence illumination (60/s; Strobex 236, Chadwick Helmuth, Mountain View, CA, USA; and filter block Zeiss 9). After recording, the resulting video was digitalised to MPEG format using Thyphoon DVD maker and TVR software (ANUBIS Electronic Ltd., Kowloon, Hong Kong).

### Statistical analysis

Data are presented as the mean±s.e. For *in vitro* experiments, Student's *t*-test was used to evaluate the differences between groups. For *in vivo* experiments, Mann–Whitney *U*-test was used to evaluate the differences between groups. In both calculations, *P*<0.05 was considered statistically significant.

## Results

### Isolation and expansion of human MSC

Bone marrow aspirates were obtained from normal human donors, isolated, and expanded according to our recent publication (Kallifatidis *et al*, 2008). Cells had a typical spindle shape, consistent with the morphology reported by others (Pittenger and Marshak, 2001). Although MSC do not have a specific antigen profile, we verified for each culture that isolated cells were negative for typical haematopoietic antigens CD45, CD34, and CD38 and were positive for CD44 and CD105 (data not shown). The doubling time of our cultures varied between 30 and 40 h, and cells could be expanded to 8–12 passages before reaching senescence. Thus, based on available criteria, the cells used in our experiments had the properties of human MSC as described.

### Specific migration of MSC to PDGF, EGF, and VEGF

**Figure 1:**
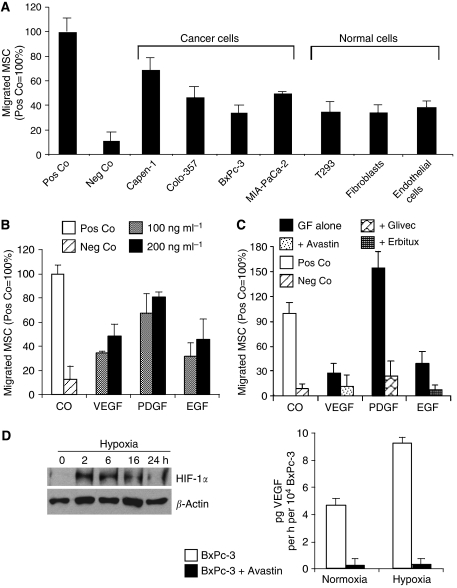
Migration of MSC to growing tumour and normal cells, VEGF, PDGF, and EGF. (**A**) Established cell lines from pancreatic cancer (Capan-1, Colo357, BxPc-3, and MIA-PaCa-2), kidney (T293), and primary cell lines from fibroblasts and endothelial cells were cultured in medium containing 2% FCS for 48 h. Supernatant was transferred to the lower well and migration of MSC placed to the upper well was measured in a ChemoTx system as described in Materials and methods. Pos Co, cell-free medium with 20% FCS; Neg Co, cell-free medium with 2% FCS. (**B**) Dose-dependent migration of MSC towards medium containing 2% FCS alone (CO) or to VEGF, PDGF, and EGF in 2% FCS and in concentrations indicated. (**C**) Migration of MSC to growth factors alone (GF alone) or to growth factors in the presence of the inhibitor of PDGF receptor (Glivec, 3 *μ*M), or blocking antibodies to EGF receptor (Erbitux, 3 *μ*M), or VEGF (Avastin, 25 *μ*g/ml). (**D**) Induction of HIF-1*α* and secretion of VEGF by pancreatic cancer cells following hypoxia. For the induction of hypoxia, the pancreatic cancer cell line BxPc-3 was treated with CoCl_2_ (100 *μ*M). Two to 16 h later, protein expression of HIF-1*α* was examined by western blot analysis. *β*-Actin served as a control for equal conditions. For the evaluation of VEGF secretion, a six-well plate with growing BxPc-3 cells was placed in a modular incubator chamber and hypoxia was induced by floating with a preanalysed air mixture (89.25% N_2_, 10% CO_2_, 0.75% O_2_) at 37°C for 16 h. Immediately thereafter, VEGF secretion into the supernatant was analysed by the ELISA assay, as described in Materials and methods. Results presented are from three independent experiments and s.d. are shown.

Factors released by cancer cells may be potential mediators of MSC migration. To test this hypothesis, we performed *in vitro* migration assays using Transwell plates to evaluate the tropism of human MSC for cancer cells. We first investigated if human established pancreatic cancer cell lines were capable of stimulating the migration of MSC. Normal cells, such as T293, primary fibroblasts, and endothelial cells, were also investigated. Mesenchymal stem cells were placed in the upper wells, and conditioned medium from cells grown in medium with 2% FCS was placed in the lower wells. Cell-free medium with 20 or 2% FCS was used as positive and negative controls, respectively. A semiporous membrane (12 *μ*m pores) separated the wells. Migration was quantified by directly visualising and counting migrated cells under the microscope after cell staining. Exposure to cell-free medium with 20% FCS and to all cell-conditioned media resulted in significant migration of MSC, when compared with cell-free medium with 2% FCS (Figure 1A). The observed differences in migration were not due to the increase in MSC proliferation because the total number of MSC (migrating plus nonmigrating) was the same for each condition. As 20% FCS and conditioned medium from growing cells stimulated significant MSC migration, it is plausible that growth factors may be chemoattractants for MSC. Therefore, to analyse the role of growth factors in MSC migration, PDGF, EGF, or VEGF was added to the lower wells. Maximal MSC migration occurred with exposure to PDGF. Intermediate levels of migration were observed after exposure to EGF followed by VEGF, which had a significant effect compared with control medium with 2% FCS (Figure 1B). To document that the increase in migration of MSC was specifically due to the presence of growth factors, inhibitors of PDGFR (Glivec), EGFR (Erbitux), and VEGF (Avastin) were added together with growth factors. These blocking substances prevented the activity of the respective growth factors (Figure 1C), suggesting specific migration of MSC to growth factors. As migration of MSC towards VEGF is the link to tumour angiogenesis, we examined VEGF expression by pancreatic cancer cells. Because VEGF is under the control of the transcription factor HIF-1*α*, which is induced by tumour hypoxia, we performed western blot experiments using BxPc-3 cells grown under normoxic and hypoxic conditions. Strong induction of HIF-1*α* was observed as early as 2 h after hypoxia, which lasted for 16 h and dropped down to basal levels after 24 h (Figure 1D). In parallel, BxPc-3 cells secreted VEGF into the supernatant, which could be completely blocked by adding Avastin to the cell culture medium as measured by an ELISA assay. Thus, it appears that enhanced levels of VEGF and other growth factors secreted by pancreatic cancer cells under hypoxic conditions lead to the migration of MSC.

### MSC are attracted by reconstructs of tumour blood vessels

**Figure 2:**
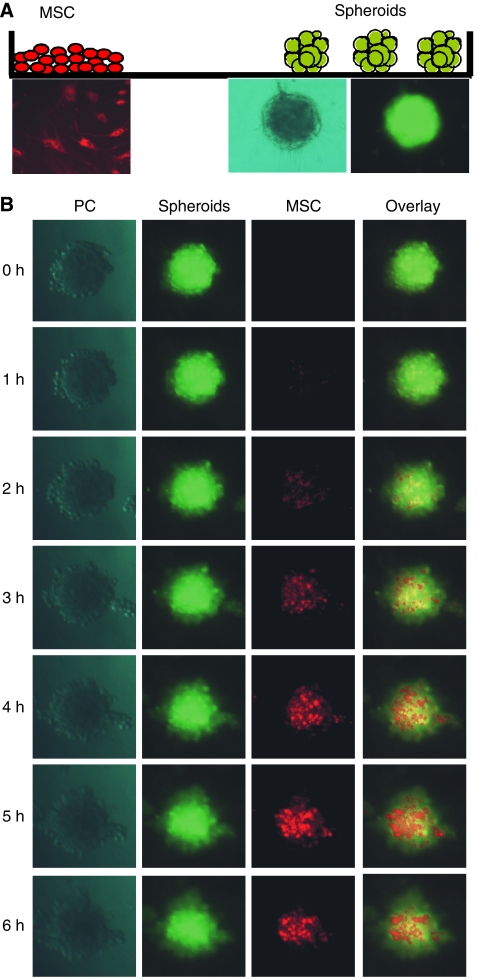
Migration of MSC to spheroids of pancreatic tumour cells. (**A**) Green-labeled spheroids (composed of 5 × 10^2^ MIA-PaCa-2, 2.5 × 10^2^ human primary fibroblasts, and 2.5 × 10^2^ HUVEC) and red-labeled MSC were seeded in opposite edges of a well of a 24-well plate and covered with methocel/collagen solution as described in Materials and methods. (**B**) Cells were analysed using a fluorescence microscope (Olympus IX 70) with a red and green filter, 20-fold magnification and two-fold binning immediately after seeding. The focus was put to spheroids. Migration of red fluorescent MSC to green fluorescent spheroids was monitored by time-lapse photography during a period of 12 h and a picture was taken every 5 min. Representative data of one experiment out of three similar is shown.

To examine, whether MSC may be attracted to tumour blood vessel reconstructs, we created tumour cell spheroids. These consisted of MIA-PaCa-2 pancreatic cancer cells, primary fibroblasts, and HUVECs. Pancreatic cancer cells and fibroblasts are known to overexpress many growth factors (Korc, 2007), including VEGF (Sipos *et al*, 2002). This, in turn, may lead to the paracrine stimulation of PDGF expression in HUVECs as recently described (Reinmuth *et al*, 2007). Therefore, tumour cell spheroids may be strong chemoattractants for MSC. To prove this hypothesis, green fluorescent spheroids and red fluorescent MSC were seeded opposite in wells of a 24-well plate and separated by a methylcellulose/collagen solution (Figure 2A). The invasion of MSC into spheroids was documented by time-lapse microscopy over 12 h focused on the green fluorescent spheroids. Within 1 h, red MSC came into the focus, indicating that MSC migrate to the spheroids (Figure 2B). Migration was completed within 5 h. These results suggest the migration of MSC to pancreatic tumour spheroids.

### MSC induce sprouting but do not differentiate into endothelial cells *in vitro*

**Figure 3:**
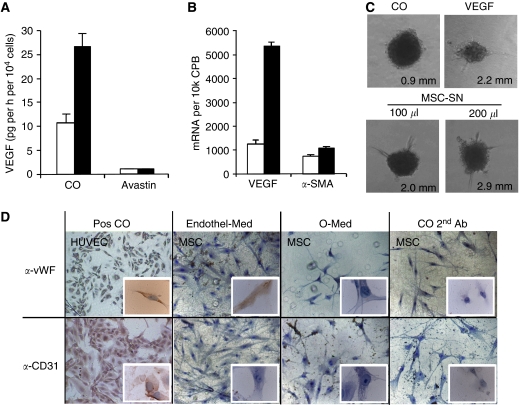
Mesenchymal stem cells induce sprouting of endothelial cells by VEGF expression but do not differentiate into endothelial cells. (**A**) Vascular endothelial growth factor protein in supernatant of MSC cultured under hypoxic (black bars) or normoxic (white bars) conditions in the presence or absence of Avastin (25 *μ*g ml^−1^) was analysed by the ELISA assay. (**B**) RNA expression of VEGF was analysed by RT-PCR in MSC. The expression of *α*-SMA served as control for equal conditions. (**C**) A volume of 100 or 200 *μ*l supernatant from MSC (as indicated), which have been cultured in medium containing 2% FCS for 48 h or recombinant VEGF (50 ng ml^−1^), was transferred to spheroids consisting of 1 × 10^3^ HUVEC growing in wells of a 24-well plate 48 spheroids per well. The length of sprouts was analysed by cell^B^ 2.3 software and median lengths are given. Representative spheroids have been visualised by phase-contrast microscopy using an Olympus CKX41 microscope, × 10 magnification, and a colorview camera soft imaging system. (**D**) Mesenchymal stem cells were seeded on chamber slights covered with fibronectin and cultured in endothelial cell medium containing VEGF (50 ng ml^−1^) for 2 weeks. Immunohistochemistry was performed using specific antibodies for the detection of the specific endothelial cell markers von-Willebrand factor (*α*-vWF) and CD31 (*α*-CD31). Human umbilical vein endothelial cells were used as positive control. A Leica DMRB microscope and × 1000 magnification were used.

To investigate the effects of paracrine factors secreted by MSC on angiogenesis, we measured VEGF expression in MSC cultured under normoxic and hypoxic conditions. The secretion of VEGF in cell culture supernatant was analysed by the ELISA assay and the expression of RNA by RT-PCR. By this way, we found that MSC express a basal level of VEGF protein and RNA, which could be enhanced by hypoxia (Figure 3A and B). Total blocking of basal and induced VEGF expression by Avastin served as control. To see whether the MSC-secreted VEGF is able to contribute to angiogenesis, we added MSC supernatant to HUVEC. This resulted in strong induction of sprouting in similar intensity as observed with the addition of recombinant VEGF alone (Figure 3C). Owing to this obvious angiogenic potential of MSC, we examined the differentiation of MSC into endothelial cells by culturing the MSC for 1, 2, or 3 weeks in endothelial cell culture medium containing VEGF. However, even after prolonged time, we could detect the expression of typical endothelial cell markers, such as CD31 or von Willebrand factor only in very few (about 0.01%) MSC (Figure 3D). In contrast, HUVECs, which were used as positive control, strongly expressed these typical markers for endothelial cells. However, 10% of MSC from the same fraction kept their differentiation potential to osteocytes and adipocytes as tested by culture in specific differentiation media as described (data not shown). Therefore, the differentiation of MSC into endothelial cells may be neglected as a main factor responsible for an angiogenic potential of MSC.

### Migration of MSC in tumour blood vessels and attachment to vessel endothelium

**Figure 4:**
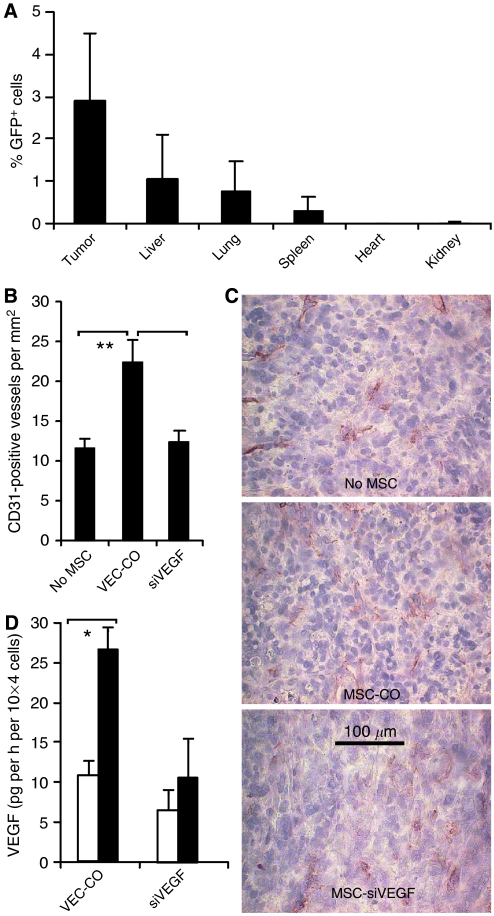
Migration of MSC in orthotopic MIA-PaCa-2 pancreatic xenografts in nude mice and the incorporation in tumour blood vessels. (**A**) Four mice with MIA-PaCa-2 orthotopic pancreatic cancer xenografts were injected with lentiviral eGFP-labeled MSC (4 × 10^5^ in tail vein). Four xenografted mice received PBS injection and served as controls. Three days after injection, mice were killed followed by the resection of organs and xenografts. Cells were isolated from tissue pieces and examined by flow cytometry for expression of green fluorescence of eGFP-expressing MSC. Mean bars±s.e. are shown. (**B**) Four xenografted mice per group were injected with PBS only (no MSC) or with MSC transduced with lentiviral control vector (VEC-CO), or with MSC transduced with lentiviral siRNA towards VEGF (siVEGF). Three days later, microvessel density in cryosections of xenografts was analysed by immunohistochemistry for CD31 in a Leica DMRB microscope with 250-fold magnification. Microvessel density was quantified using eight images from each of four different tumours per group. Microvessels per field of 1 mm^2^ were counted. (**C**) Data from one representative staining per group are shown. (**D**) Vascular endothelial growth factor protein in supernatant of MSC transduced with lentiviral control vector (VEC-CO) or with siRNA towards VEGF (siVEGF) cultured under normoxic (white bars) or hypoxic (black bars) conditions was analysed by the ELISA assay. Statistical significance was determined by *t*-test (*P*<0.05) and is indicated by an asterisk.

Mice with human orthotopic pancreatic cancer xenografts were injected with LV-transduced MSC (4 × 10^5^ in tail vein) conferring the expression of enhanced green fluorescent protein (eGFP). Xenografted mice, which did not receive MSC, served as controls. Three days later, mice were killed followed by resection of xenografts and organs. Isolated cells were examined by flow cytometry for eGFP expression (Figure 4A). The highest eGFP expression was observed in tumour xenografts, but a minor percentage was also detected in normal organs, such as the lung, spleen, and kidney but not in the liver or heart. Weak green fluorescence was observed in tumours of mice that did not receive MSC, suggesting autofluorescence of the mouse tissue. This result indicates a tumour-specific migration of MSC and demonstrates that few MSC reside in other organs according to a natural function of MSC in tissue remodelling (Jiang *et al*, 2002). To see whether MSC influence tumour angiogenesis, we injected MSC with lentiviral control vector or with lentiviral siRNA into mice with orthotopic MIA-PaCa-2 xenografts. Three days later, cryosections of xenografts were examined for the expression of CD31 to detect blood vessels. To quantify the density of blood vessels, we counted CD31^+^ structures per high-power field ( × 250) (Figure 4B and C). By this way, we found that MSC with empty vector lead to a doubling of blood vessels in contrast to mice that received no MSC or MSC with siRNA-inhibited VEGF expression. As siVEGF lead to a strong inhibition of VEGF protein secretion in transduced MSC (Figure 4D), the production of VEGF by MSC may be a major reason for the angiogenic capacity of MSC.

### Intravital microscopy of MSC recruitment in mice

**Figure 5:**
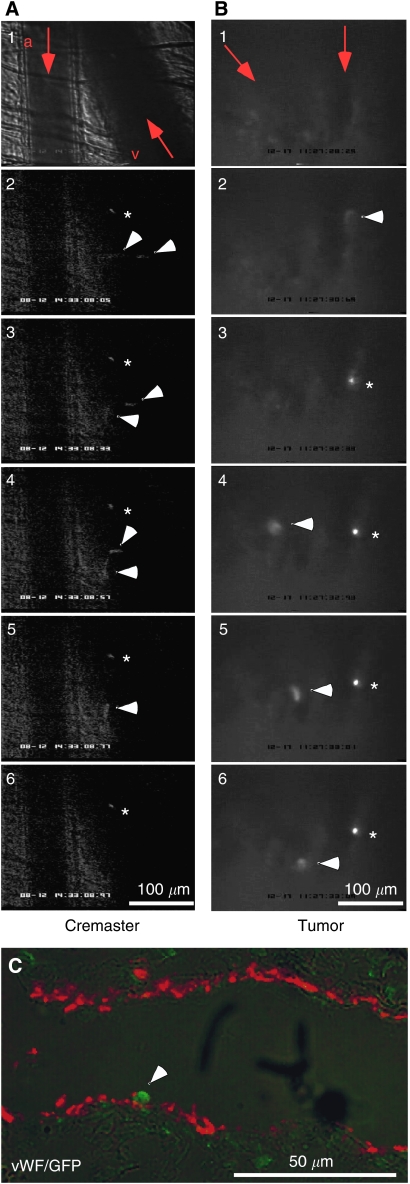
Recruitment of MSC in mice by intravital microscopy reveals attachment to normal and tumour blood vessels. Mice with MIA-PaCa-2 orthotopic pancreatic cancer xenografts were injected with lentiviral GFP-transduced MSC (four consecutive injections of 10^5^ MSC per 0.2 ml normal saline in time intervals of about 15 min) into the carotid artery. Photographs of circulating MSC in (**A**) cremaster muscle and (**B**) tumour vessels were taken and analysed as described in Materials and methods. Circulating (white arrow) and attached (white asterisk) green fluorescent MSC appeared during the first minute after injection. Red arrows indicate the direction of flow. a, artery; v, venule. Pictures are freezed images from video sequences provided in the supplement. (**C**) Immunofluorescence staining of a GFP-expressing MSC incorporated in vWF-positive endothelial cells of a vessels was detected in an orthotopic pancreatic xenograft of nude mice, which have been injected with MSC as described in Figure 4.

To further elucidate the process of MSC homing *in vivo*, we monitored microcirculation in cremaster muscle and in orthotopic Mia-PaCa-2 xenograft tumours using fluorescence intravital microscopy immediately after intraarterial injection of MSC expressing eGFP. Within 5–10 s after the injection of MSC, the green fluorescent cells appeared in the inflamed cremasteric microcirculation, first freely flowing in small arteries and arterioles, and later in the capillary network where some of them got already stuck. As soon as 1 min after MSC administration, eGFP^+^ cells were detectable in the cremasteric venular tree (Figure 5A, Supplementary Figure S1). We observed firm arrest of individual MSC in cremasteric venules and veins (vessel diameter 120 *μ*m, centerline blood flow velocity of 2100 *μ*m s^−1^ with a resulting wall shear rate of 700 s^−1^). Although technically more challenging, we were able to observe MSC homing in the xenograft tumours similar to MSC recruitment in cremaster muscle. After arterial circulation, some MSC adhered in tumour capillaries and later also in tumour venules and veins (vessel diameter 45 *μ*m, centerline blood flow velocity of 800 *μ*m/s with a resulting wall shear rate of 700 s^−1^) (Figure 5B, Supplementary Figure S2). A few of the observed MSC detached 30 s after initial arrest. The attachment of MSC to vascular endothelium was confirmed by immunofluorescence staining of an eGFP-expressing MSC within vWF-positive endothelial cells within a tumour vessel of a pancreatic cancer xenograft (Figure 5C).

## Discussion

Cellular therapy with human MSC has great potential for use in regenerative medicine and is currently in clinical development. Mesenchymal stem cells are being investigated in the treatment of bone and cartilage defects, and injured myocardium after acute infarction (Giordano *et al*, 2007). The ability of MSC to differentiate into several lineages of connective tissue is well documented (Prockop, 1997). Recent evidence has suggested that MSC may also differentiate into endothelial and vascular smooth muscle cells (Oswald *et al*, 2004). In this report, we set out to clarify molecular mechanisms of MSC migration towards tumour blood vessels and the cellular fate of implanted MSC in participating in vasculogenesis *in vivo*. Our study shows the migration of MSC towards growth factors secreted by growing tumour and normal cells, with PDGF as most potent chemoattractant, followed by EGF and VEGF, in line with recent findings (Nakamizo *et al*, 2005; Schmidt *et al*, 2006a). Moreover, we found the secretion of VEGF by MSC itself under basal conditions and the enhancement of VEGF mRNA and protein secretion into the supernatant by hypoxia. As expected, the transfer of supernatant from MSC to HUVEC increased sprouting, supporting the angiogenic potential of MSC. Therefore, VEGF production by MSC may be a crucial factor responsible for an angiogenic potential of MSC. Another reason for contribution of MSC to angiogenesis may be the differentiation of MSC in endothelial cells as recently suggested (Oswald *et al*, 2004; Silva *et al*, 2005; Song *et al*, 2007). As these data are unclear, we tried to differentiate MSC into endothelial cells by culturing them in endothelial cell culture medium with VEGF. However, we found the differentiation of only few MSC below 0.01% into cells expressing vWF, a typical marker of vascular endothelial cells. The majority of MSC remained undifferentiated under these conditions, although the differentiation potential into adipocytes and osteocytes was intact in the same fractions. The reason for this low differentiation capacity into endothelial cells may be of experimental nature due to suboptimal growth conditions for MSC in endothelial cell culture medium. After 3 weeks in this medium, the MSC resembled rather senescent cells than endothelial cells. However, the differentiation capacity of MSC into endothelial cells is underlined by other reports. In 1999, Barry *et al* (1999) identified the endothelial marker endoglin (CD105) in BM-derived MSC grown on an endothelial cell-specific membrane. Although undifferentiated BM-derived MSC did not express vWF (Hu *et al*, 2003), expression of this and some molecules specific to endothelial cells was found after endothelial differentiation on the cell surface of MSC (Oswald *et al*, 2004; Sun *et al*, 2005). But blood vessels are not only composed of endothelial cells. Endothelial cells constitute the inner lining of the vessel wall, and pericytes and vascular smooth muscle cells (summarised as mural cells) envelop the surface of the vascular tube in mature, stable vessels. During the early stage of angiogenesis, neovascular sprouts are composed primarily of endothelial cells and are subsequently stabilised by recruiting mural cells (Jain, 2003). Human MSC implanted in immunodeficient mice recruit into blood vessels as shown by our immunohistochemistry and microcirculation studies. However, we did not detect *in vivo* differentiation of MSC into endothelial cells and the function of MSC in endothelium is unclear so far. In related models, it has been described that co-implantation of HUVEC with a murine mesenchymal precursor cell line (10T1/2) that differentiates into mural cells is necessary for the formation of long-lasting blood vessels (Koike *et al*, 2004). In consequence, MSC may be a source for mural cells or be involved in recruitment of them. A recent publication points to this direction, as co-implantation of HUVEC and human MSC in immunodeficient mice was able to form human mature blood vessels, which are stable and functional for more than 3 months (Sanz *et al*, 2008). Another recent study suggested MSC as perivascular cell precursors and MSC seem to contribute to a functional vasculature by differentiation into pericytes (Au *et al*, 2008). *In vivo*, we detected homing of MSC to tumour blood vessels of an orthotopic pancreatic carcinoma xenograft in immunodeficient mice. After injection of MSC in tail vein, we found enhanced vessel density by control MSC but not by MSC expressing siRNA directed towards VEGF. Therefore, our data suggest that VEGF production by MSC is the critical factor mediating the angiogenic potential of MSC. Tumour hypoxia, a feature typical for pancreatic cancer may further increase the angiogenic effect of MSC, as our *in vitro* data show the expression of HIF-1*α* by pancreatic cancer cells under hypoxic conditions, which, in turn, leads to expression and secretion of VEGF by tumour cells. Enhanced VEGF levels may increase migration of MSC, which, after recruitment to pancreatic tumours, itself produce VEGF and contribute by this way to the hypoxia-initiated angiogenic effect.

For *in vivo* detection of MSC recruitment in trauma-induced inflammation of cremaster muscle and orthotopic MIA-PaCa-2 xenograft tumours, we used intravital microscopy. By this way, we found a very early arterial circulation of injected MSC followed by a final arrest of some MSC in the capillary network, which is likely to be due to obstruction. Other MSC were detected in the venular tree as early as 1 min after MSC administration where some of them adhered firmly to the endothelium. Only very few transiently attached MSC detached again from the vessel wall. Although there are some rolling MSC detectable, the rather sudden arrest of MSC seems to be similar to the VCAM-1-dependent fashion of leukocyte adhesion triggered by VLA-4 (Ley *et al*, 2007). As VLA-4 is known to be expressed on MSC and VCAM-1 on inflamed vascular endothelium (Ley *et al*, 2007), one may hypothesise an *in vivo* relevance of the interaction of these two molecules during MSC recruitment as supported by recent studies (Ruster *et al*, 2006; Segers *et al*, 2006). Furthermore, similar to leukocytes, MSC seem to be recruited preferentially in low flow (capillaries) or medium flow (venules) vessels providing optimal haemodynamic conditions, that is, wall shear rate (Long *et al*, 2004). With these respects to leukocyte recruitment, further elucidation of exact mechanisms of the early steps of MSC recruitment into both peripheral tissue and neoplasic tumours is required.

In conclusion, we demonstrate that human MSC from BM specifically migrate to tumour blood vessels of pancreatic carcinoma *in vitro* and *in vivo*, which can be attributed to tumour hypoxia-induced secretion of VEGF and other growth factors. We observed long-term (3 days) interaction of MSC with the endothelium of tumour blood vessels. Very few of MSC differentiated into endothelial cells *in vitro*, whereas no differentiation *in vivo* could be observed. We found high VEGF production by MSC, which was further enhanced by hypoxia. Therefore, secretion of VEGF may be the crucial factor determining the angiogenic potential of MSC. However, before these findings can be used for the creation of optimised treatment schedules in patients, one should keep in mind that the role of MSC is complex and these stem cells interact with multiple other molecules and cells. The results of our study are limited by the use of only one pancreatic cancer cell line. But cancer cells differ and further experiments are necessary to fully understand the complex function of MSC in the organism.
