# Supplementary material for: VEGF expression by mesenchymal stem cells contributes to angiogenesis in pancreatic carcinoma
Source: Br J Cancer. 2008 Jul 29;99(4):622–31. doi: 10.1038/sj.bjc.6604508 (PMC2527820; doi:10.1038/sj.bjc.6604508)
Supplement: Supplementary Figure Legends [file 6604508x3.doc]

**Supplemental Figure Legend**

**Supplemental Videos S1 and S2:** Recruitment of MSC one minute after injection of 10^5^ MSC in mice was examined by intravital microscopy according to Fig. 5. Supplemental Video S1: Recruitment of GFP-MSC in cremaster muscle vessels. Supplemental Video S2: Recruitment of GFP-MSC in an orthotopic pancreatic xenograft tumor vessels (MIA-PaCa2).
